# Supplementary material for: Analysis of PPARγ Signaling Activity in Psoriasis
Source: Int J Mol Sci. 2021 Aug 10;22(16):8603. doi: 10.3390/ijms22168603 (PMC8395241; doi:10.3390/ijms22168603)
Supplement: Supplementary file 1 [file ijms-22-08603-s001.zip › Supplemental materials_Analysis of PPARg signaling activity in psoriasis/Pathway models/Models images and html files/PPARG inhibits phenotypes assotiated with psoriasis/125645.html]

amyloidosis


# Disease amyloidosis

|  |  |
| --- | --- |
| URN | urn:agi-meshdis:Amyloidosis |
| Total Entities | 14 |
| Connectivity | 1815 |
| Name | amyloidosis |

---

|  |  |
| --- | --- |
| ChildConcepts | amyloid neuropathy |
|  | familial amyloidosis |
|  | Muckle-Wells syndrome |
|  | cardiac amyloidosis |
|  | renal amyloidosis |
|  | vascular amyloidosis |
|  | AA amyloidosis |
|  | ATTR amyloidosis |
|  | Abeta2M amyloidosis |
|  | familial Mediterranean fever |
|  | lichen amyloidosis |
|  | primary amyloidosis |
|  | cutaneous amyloidosis |
|  | macular amyloidosis |

---

|  |  |
| --- | --- |
| Pathway | Complement Cascade Activation by Pentraxins |
|  | PPARG inhibits phenotypes assotiated with psoriasis |

---

|  |  |
| --- | --- |
| MedScan ID | 9010051 |

---

|  |  |
| --- | --- |
| Alias | paraamyloidosis |
|  | Amyloid Disease |
|  | systemic amyloidoses |
|  | amyloid tumor |
|  | amyloidoma |
|  | amyloid tumors |
|  | senile amyloidosis |
|  | beta fibrillosis |
|  | amyloidomas |
|  | Amyloidoses |
|  | amyloidotic |
|  | Amyloidosis |
|  | senile amyloidoses |
|  | Amyloid Diseases |
|  | beta fibrilloses |
|  | systemic amyloidosis |

---

|  |  |
| --- | --- |
| MeSH ID | D000686 |
|  | M0001053 |

---

|  |  |
| --- | --- |
| MeSH Heading | Amyloidosis |

---

|  |  |
| --- | --- |
| Orphanet ID | 69 |
|  | 85446 |

---
